# Utility of Inflammatory Markers in Detection of Perioperative Morbidity After Laparoscopic Sleeve Gastrectomy, Laparoscopic Roux-en-Y Gastric Bypass, and One-Anastomosis Gastric Bypass**—**Multicenter Study

**DOI:** 10.1007/s11695-020-04636-8

**Published:** 2020-04-28

**Authors:** Michał Wysocki, Piotr Małczak, Mateusz Wierdak, Maciej Walędziak, Hady Razak Hady, Inna Diemieszczyk, Monika Proczko-Stepaniak, Michał Szymański, Natalia Dowgiałło-Wnukiewicz, Jacek Szeliga, Michał Pędziwiatr, Piotr Major

**Affiliations:** 1 2nd Department of General Surgery, Jagiellonian University Medical College, Jakubowskiego 2 St., 30-688 Cracow, Poland; 2 Department of General, Oncological, Metabolic and Thoracic Surgery, Military Institute of Medicine, Warsaw, Poland; 3 First Department of General and Endocrine Surgery, Medical University of Bialystok, Bialystok, Poland; 4 Department of General, Endocrine and Transplant Surgery, Medical University of Gdańsk, Gdańsk, Poland; 5 Department of General, Minimally Invasive and Elderly Surgery, University of Warmia and Mazury, Olsztyn, Poland; 6 Department of General, Gastroenterological, and Oncological Surgery, Collegium Medicum Nicolaus Copernicus University, Torun, Poland

**Keywords:** Laparoscopic sleeve gastrectomy, Laparoscopic Roux-en-Y gastric bypass, One-anastomosis gastric bypass, CRP, Inflammatory markers, Morbidity, Mortality

## Abstract

**Background:**

The most commonly performed bariatric operations are laparoscopic sleeve gastrectomy (LSG) and bypass surgeries (laparoscopic one-anastomosis gastric bypass (OAGB) and laparoscopic Roux-en-Y gastric bypass (LRYGB)), and predicting perioperative morbidity is crucial for early, safe patient discharge. We aimed to determine whether C-reactive protein (CRP) and white blood count (WBC) measured on the first postoperative day predicts perioperative morbidity in the first 30-days after LSG and bypass surgeries.

**Methods:**

We retrospectively analyzed data for 1400 patients who underwent bariatric surgery in seven bariatric centers from 2014 to 2018. Patients were divided into a complicated group (patients with postoperative complications) and a non-complicated group. We also performed separate analyses for LSG and bypass surgeries.

**Results:**

Patients were 929 women (66%) and 471 men (34%) with a median age of 42 years (range, 35–51 years); 1192 patients underwent LSG (85%), 120 underwent LRYGB (9%), and 80 underwent OAGB (6%). We performed ROC analyses to set cut-off points, followed by multivariate logistic regressions. CRP > 33.32 mg/L increased the odds ratio (OR) of perioperative complications after LSG 2.27 times, while WBC > 12.15 × 10^3^/μL on postoperative day 1 was associated with a 3.34-times greater or of developing complications. WBC > 13.78 × 10^3^/μL was associated with a 13.34-times higher or of perioperative morbidity in patients undergoing bypass surgeries.

**Conclusion:**

Even slightly elevated CRP and WBC on postoperative day 1 should alert surgeons to the potential risk of perioperative morbidity.

## Introduction

The number of bariatric procedures is increasing annually. According to the 2016 International Federation for the Surgery of Obesity and Metabolic Disorders (IFSO) survey data from 58/62 IFSO Societies, the total number of bariatric/metabolic procedures performed in 2016 was 685,874. The most common primary surgical bariatric/metabolic procedure was laparoscopic sleeve gastrectomy (LSG) (340,550; 53.6%), followed by Roux-en-Y gastric bypass (LRYGB) (191,326; 30.1%), and one-anastomosis gastric bypass (OAGB) (30,563; 4.8%) [1, 2]. While the benefits of bariatric surgery are unquestionable, namely, weight loss and remission of comorbidities, surgeons must consider the risk of complications, especially because bariatric surgery is an elective procedure. The perioperative morbidity for all Clavien–Dindo complication grades after bariatric surgery is relatively low at 10.1%, with treatment according to the Enhanced Recovery After Bariatric Surgery (ERABS) protocol [3]. Once complications occur, they are an issue for both the patient and the surgeon. Length of hospital stay (LOS) after bariatric surgery is ≤ 3 days, which limits close patient monitoring postoperatively [4]. Therefore, it is particularly important to identify specific markers for early detection of complications. In centers with implemented ERAS and ERABS protocols, discharges from hospital usually occur at postoperative day (POD) 2, sometimes at POD 1 [3, 5]. The goal of this study was to find early markers, even nonspecific, i.e., without highest sensitivity and specificity possible. Those markers would indicate patients, who potentially develop perioperative morbidity and who should have more attention and probably should stay longer at hospital. Inflammatory marker measurement may achieve this goal and has met similar targets in several surgical fields [6–10]. We aimed to determine the correlations between C-reactive protein (CRP) and white blood count (WBC), both measured routinely on the first POD, and perioperative morbidity in the first 30 days after LSG, OAGB, and LRYGB.

## Materials and Methods

### Methods

This was a retrospective cohort study analyzing data for patients who underwent surgical treatment for morbid obesity in seven referral bariatric centers from February 2014 to March 2018. Each participating center provided specific data, which were processed and used in the overall analysis. The study was designed and performed according to the STROBE guidelines for observational studies [11]. The included patients underwent LSG, LRYGB, or OAGB, which are the most commonly performed bariatric procedures in these centers. Exclusion criteria were revision surgeries; bariatric operations other than LSG, LRYGB, or OAGB; extended surgeries during which other procedures were performed; and lack of necessary data or patients lost to follow-up. All bariatric operations in the participating centers were performed laparoscopically using comparable surgical techniques, and perioperative care was performed according to enhanced recovery after surgery protocols (ERAS® [3, 5, 12]), which ensured reliable data comparison. Part of our study was a review of the medical records from the databases of the participating centers. Baseline patients’ characteristics were sex, age, body mass index, relevant comorbidities, and the bariatric procedure.

The primary endpoint was determining the usefulness of routinely tested inflammatory markers on POD 1, i.e., CRP and WBC, to predict postoperative morbidity after LSG, LRYGB, and OAGB.

Patients were divided into two groups: a complicated group constituting patients with postoperative complications, and a non-complicated group constituting patients without postoperative complications. Additionally, patients’ data were analyzed separately for LSG and gastric bypass (LRYGB and OAGB).

We defined postoperative morbidity as any deviation from the standard postoperative course requiring additional adequate treatment measures within 30 days of the initial procedure classified according to the Clavien–Dindo grading system [13]. LOS was defined as the period from surgery to discharge. Prolonged LOS was defined as > 4 days because, depending on the treatment protocols in participating centers, planned hospitalization time for bariatric surgery was 3 or 4 days. Readmissions were analyzed only if they occurred in the index hospitals.

CRP and WBC were measured at each participating center on POD 1 in hospital laboratories with ISO 9001 certificates, using comparable laboratory methods. CRP was measured in blood serum using an immunonephelometric technique, and WBC was measured in full blood samples preserved with ethylenediaminetetraacetic acid, using flow cytometry.

### Statistical Analysis

Statistical analyses were performed using Statistica 13.5 software (StatSoft®, Tulsa, OK). Continuous values were presented as means with standard deviations or medians with interquartile ranges, as appropriate. Quantitative variables were compared using Student’s *t* test or the Mann–Whitney *U* test, while qualitative variables were compared with the chi-squared test with or without Yates’ correction. Optimal cut-off points for laboratory parameters were chosen using Youden’s index. Multivariate logistic regression models adjusted for relevant intergroup baseline differences were performed to calculate odds ratios (ORs) with 95% confidence intervals (CIs). *P* values ≤ 0.05 were considered statistically significant.

### Study Group

**Fig. 1:**
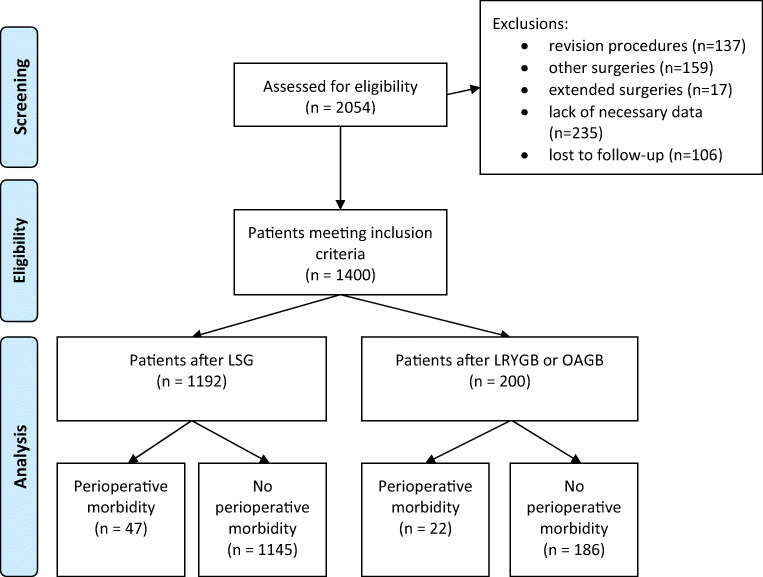
Flow chart of patients through the study

The study group constituted 1400 patients from seven bariatric centers with complete data for a 30-day postoperative follow-up. Figure 1 is a patient flow chart. There were 929 women (66%) and 471 men (34%) in the study group, with a median age of 42 years (range, 35–51 years); 1192 patients underwent LSG (85%), 120 underwent LRYGB (9%), and 80 underwent OAGB (6%).

## Results

### Group Characteristics

Perioperative morbidity was encountered in 69 patients (4.93%), and morbidity rates differed significantly between LSG and bypass surgery (*p* < 0.001). Mortality was 0.29% (four patients). The general characteristics of the study groups and differences in perioperative laboratory results are presented in Table 1.

**Table 1:** General characteristics patients after LSG or LRYGB and OAGB

	Complicated	Non-complicated	p value
LSG			
*n* (%)	47 (3.94%)	1145 (96.06%)	n/a
Males/females, *n* (%)	14/33 (30%/70%)	402/743 (35%/65%)	0.453
Median age, years (IQR)	40 (35–49)	41 (34–50)	0.944
Median BMI, kg/m^2^ (IQR)	44.46 (39.31–49.84)	43.59 (40.27–48)	0.435
Diabetes mellitus type 2, *n* (%)	13 (27.66%)	251 (21.96%)	0.357
Arterial hypertension, *n* (%)	28 (59.57%)	575 (50.22%)	0.211
Obstructive sleep apnea, *n* (%)	7 (14.89%)	147 (12.84%)	0.637
Median operative time, min (IQR)	75 (60–105)	70 (55.5–90)	0.113
Median LOS, days (IQR)	5 (4–7)	3 (3–4)	< 0.001
Readmissions, *n* (%)	12 (25.53%)	9 (0.79%)	< 0.001
Median CRP on POD 1, mg/L (IQR)	28.77 (15.1–48.8)	18.84 (11.6–29.6)	0.002
Median WBC on POD 1, × 10^3^/μL (IQR)	12.42 (10.7–16.83)	11.16 (9.25–13.21)	0.002
Bypasses			
*n* (%)	22 (10.58%)	186 (89.42%)	n/a
Males/females, *n* (%)	9/13 (41%/59%)	46/140 (25%/75%)	0.088
Median age, years (IQR)	44 (40–50)	46 (39–54)	0.188
Median BMI, kg/m^2^ (IQR)	42.05 (37–46.60)	42.05 (38.30–46.66)	0.857
Diabetes mellitus type 2, *n* (%)	11 (50.0%)	42 (22.58%)	*0.022*
Arterial hypertension, *n* (%)	14 (63.64%)	112 (60.22%)	0.756
Obstructive sleep apnea, *n* (%)	4 (18.18%)	34 (18.28%)	0.965
Median operative time, min (IQR)	95 (65–150)	95 (65–150)	0.810
Median LOS, days (IQR)	4 (4–8)	3 (2–4)	*0.003*
Readmissions, *n* (%)	7 (31.82%)	5 (2.69%)	< 0.001
Median CRP on POD 1, mg/L (IQR)	13.51 (6.7–27.76)	18.68 (10–41.1)	0.316
Median WBC on POD 1, × 10^3^/μL (IQR)	15.69 (13.88–16.78)	11.28 (9.47–13.23)	< 0.001

*BMI*, body mass index; *IQR*, interquartile range; *LOS*, length of hospital stay; *CRP*, C-reactive protein; *WBC*, white blood count; *POD*, postoperative day

### Main Outcomes

Perioperative morbidity data after LSG and bypass surgery are presented in Table 2.

**Table 2:** Perioperative morbidity sorted out regarding Clavien–Dindo classification of surgical complications

C–D	Perioperative	LSG	LRYGB and OAGB
V	Urosepsis	1	0
	GI leak	1	0
	Intra-abdominal hernia, GI leak	0	1
	Acute pancreatitis, GI leak, peritonitis	1	0
IVb	Acute respiratory failure	1	0
IVa	Heart failure	1	0
IIIb	Subphrenic abscess	2	1
	GI leak	5	3
	Postoperative bleeding (reoperation)	11	3
	GI stricture	0	2
IIIa	GI stricture	1	1
	Postoperative bleeding (treated endoscopically)	4	7
	Surgical site infection	3	1
	Intraperitoneal fluid collection	1	0
	Subphrenic hematoma, acute pancreatitis	1	0
II	Postoperative bleeding (treated with blood transfusions)	2	3
	Renal insufficiency	1	0
	*Clostridium difficile* infection	1	0
	Superior mesenteric vein and/or portal vein thrombosis	2	0
	Pneumonia	2	0
I	Vomiting/dehydration	6	1
Total		47 (3.94%)	22 (10.58%)

Regarding the cut-off points for CRP and WBC for perioperative complications, we determined these separately for LSG and bypass surgery. We were able to determine the cut-off points for all analyzed inflammatory markers for LSG for general morbidity and morbidity of III–V Clavien–Dindo grades; however, for LRYGB and OAGB, we were able to determine a significant cut-off for only WBC on POD 1 (Table 3).

**Table 3:** Results of ROC analyses of CRP on POD 1 (mg/L) and WBC on POD 1 (× 10^3^/μL) for LSG and bypasses

		Cut-off point	AUC (95% CI)	p value	Sensitivity (%)	Specificity (%)
LSG						
Morbidity	CRP	33.32	0.66 (0.56–0.76)	0.002	47.1	80.2
	WBC	12.15	0.65 (0.56–0.74)	0.001	48.7	66.9
Morbidity of III–V Clavien–Dindo	CRP	35.00	0.59 (0.46–0.72)	0.048	36.4	81.5
	WBC	12.20	0.69 (0.58–0.579)	0.001	66.7	63.3
Bypasses						
Morbidity	CRP	82.75	0.42 (0.26–0.58)	0.332	n/a	n/a
	WBC	13.78	0.8 (0.69–0.90)	< 0.001	81.8	79.6
Morbidity of III–V Clavien–Dindo	CRP	92.7	0.47 (0.25–0.69)	0.778	n/a	n/a
	WBC	13.78	0.79 (0.66–0.92)	< 0.001	85.7	77.3

The cut-off points and other possible risk factors for perioperative morbidity after LSG were analyzed in logistic regression models and are presented in Table 4. Factors that were significant in the univariate logistic regression models were selected for multivariate analyses. CRP > 33.32 mg/L and WBC > 12.15 × 10^3^/μL on POD 1, and longer operating time with LSG, were prognostic factors for LSG-related morbidity (Table 4).

**Table 4:** Logistic regression models of risk factors for perioperative morbidity after LSG

	OR	95% CI	*p* value
Univariate			
Females	1.13	0.82–1.55	0.454
Age, every year	1.00	0.97–1.03	0.975
BMI, every kg/m^2^	1.02	0.98–1.07	0.268
Diabetes mellitus type 2	1.17	0.84–1.62	0.358
Arterial hypertension	1.21	0.90–1.63	0.213
Obstructive sleep apnea	1.10	0.73–1.67	0.638
Operative time, every min	1.01	1.00–1.02	0.034
CRP on POD 1 > 33.32 mg/L	3.19	1.57–6.49	0.001
WBC on POD 1 > 12.15 × 10^3^/μL	2.20	1.15–4.21	0.018
Multivariate			
Operative time, every min	1.02	1.00–1.03	0.016
CRP on POD 1 > 33.32 mg/L	2.27	1.02–5.02	0.043
WBC on POD 1 > 12.15 × 10^3^/μL	3.34	1.54–7.25	0.002

*OR*, odds ratio; *95% CI*, 95% confidence interval; *BMI*, body mass index; *CRP*, C-reactive protein; *WBC*, white blood count; *POD*, postoperative day

Then, the same analyses were created for perioperative morbidity of III–V Clavien–Dindo grades after LSG. WBC on POD 1 > 12.2 × 10^3^/μL, determined in the ROC analysis was the only significant risk factor for perioperative morbidity of III–V Clavien–Dindo grades after LSG (Table 5).

**Table 5:** Logistic regression models of risk factors for perioperative morbidity of III–V Clavien–Dindo after LSG

	OR	95% CI	*p* value
Univariate			
Females	1.07	0.49–2.36	0.859
Age, every year	0.99	0.96–1.03	0.714
BMI, every kg/m^2^	1.02	0.97–1.07	0.449
Diabetes mellitus type 2	1.07	0.45–2.54	0.879
Arterial hypertension	1.12	0.54–2.32	0.765
Obstructive sleep apnea	0.64	0.22–2.49	0.629
Operative time, every min	1.01	1.00–1.02	0.080
CRP on POD 1 > 35 mg/L	2.06	0.82–5.18	0.126
WBC on POD 1 > 12.2 × 10^3^/μL	2.87	1.24–6.65	0.014

*OR*, odds ratio; *95% CI*, 95% confidence interval; *BMI*, body mass index; *CRP*, C-reactive protein; *WBC*, white blood count; *POD*, postoperative day

We analyzed the cut-off points and other possible risk factors for perioperative morbidity after LRYGB and OAGB using logistic regression models, and results are presented in Table 5. In the univariate analyses, type 2 diabetes and WBC on POD 1 were significant risk factors. We found that type 2 diabetes and WBC > 13.78 × 10^3^/μL on POD 1 indicated significantly increased risk for perioperative complications after LRYGB and OAGB (Table 6).

**Table 6:** Logistic regression models of risk factors for postoperative morbidity after bypasses

	OR	95% CI	*p* value
Univariate			
Females	0.48	0.19–1.18	0.110
Age	0.97	0.93–1.02	0.190
BMI	1.00	0.94–1.07	0.913
Diabetes mellitus type 2	3.23	1.12–9.09	0.029
Arterial hypertension	1.16	0.46–2.89	0.756
Obstructive sleep apnea	1.03	0.28–3.86	0.965
Operative time	1.00	0.99–1.01	0.855
CRP on POD 1, every mg/L	1.00	0.98–1.02	0.946
WBC on POD 1 > 13.78 × 10^3^/μL	13.24	4.59–38.18	< 0.001
Multivariate			
Diabetes mellitus type 2	3.13	1.04–9.48	0.043
WBC on POD 1 > 13.78 × 10^3^/μL	13.14	4.50–38.41	< 0.001

*OR*, odds ratio; *95% CI*, 95% confidence interval; *BMI*, body mass index; *CRP*, C-reactive protein; *WBC*, white blood count; *POD*, postoperative day

In case of perioperative morbidity of III–V Clavien–Dindo grades, the only significant parameter in univariate logistic regression models was WBC on POD1 > 13.78 × 103/μL (Table 7).

**Table 7:** Logistic regression models of risk factors for perioperative morbidity of III–V Clavien–Dindo after bypasses

	OR	95% CI	*p* value
Univariate			
Females	0.63	0.20–1.97	0.419
Age, every year	0.96	0.91–1.02	0.165
BMI, every kg/m^2^	1.01	0.64–1.09	0.788
Diabetes mellitus type 2	0.45	0.14–1.50	0.193
Arterial hypertension	1.68	0.51–5.59	0.394
Obstructive sleep apnea	0.43	0.05–3.52	0.425
Operative time, every min	1.00	0.99–1.01	0.635
WBC on POD 1 > 13.78 × 10^3^/μL	12.5	3.31–47.16	< 0.001
Multivariate			

*OR*, odds ratio; *95% CI*, 95% confidence interval; *BMI*, body mass index; *CRP*, C-reactive protein; *WBC*, white blood count; *POD*, postoperative day

## Discussion

Bariatric procedures are now considered standard in general surgery in Poland. Moreover, these procedures are performed in low-volume centers and private facilities, often as a single-day admission or as medical tourism. Therefore, identifying early markers predicting perioperative morbidity is needed and relevant, as the patients are usually discharged from hospital at POD 2. We found similar research and study protocols for early markers of morbidity in other fields of general surgery, particularly in colorectal surgery, but we found no studies with a large patient cohort undergoing bariatric surgery analyzing the usefulness of CRP and WBC to predict the risk of perioperative complications, as in our study.

We evaluated patients undergoing surgery for morbid obesity in accordance with the ERAS® protocols [12]. Our patient groups were similar regarding their baseline characteristics, and our results showed that CRP and WBC values differed between patients who developed complications and those who did not. Multivariate analysis and ROC analysis showed that CRP > 33.32 mg/L on POD 1 was associated with a three times greater risk of developing complications, and that WBC > 12.15 × 10^3^/μL on POD 1 was associated with a two times greater risk in patients undergoing LSG. The only inflammatory marker that was significantly associated with a higher risk of morbidity after gastric bypass was WBC > 13.78 × 10^3^/μL on POD 1, which increased the risk by 13 times. In case of perioperative morbidity of III–V Clavien–Dindo grades, WBC on POD 1 remained indicative risk factor for perioperative morbidity after LSG, as well as bypasses.

Albanopoulos et al., in their study evaluating LSG, showed that WBC and CRP were correlated with leakage or abscess on PODs 3, 5, 7, 9, and 11 [6]. However, the main disadvantage of the study was the low number of patients with complications, which rendered the conclusions difficult to interpret. Mickevicius et al. presented results after LRYGB and showed that CRP differed significantly on POD 1 between patients with and without postoperative 30-day morbidity. This finding differs from our results regarding LRYGB, which indicated that differences in CRP levels were non-significant [14]. Villard et al. identified CRP as a specific marker, however, with very low sensitivity [15]. In their study, the authors identified CRP of 50 mg/L as a predictor of complications. In contrast, our multivariate analysis predicted complications when CRP was > 33.32 mg/L. The main advantage in our analysis is that our regression models included additional factors. Williams et al. reported even higher CRP levels at > 90 mg/L on POD 1 in patients with complications and 75 mg/L in patients without complications. Warschkow et al. suggested that CRP should be routinely measured on POD 2 after LRYGB to exclude complications, and leaks in particular [16]. The authors concluded that radiological imaging examinations for intestinal leaks could be restricted to patients with serum CRP > 229 mg/L on POD 2. Accordingly, it is difficult to determine the exact cut-off point for CRP concentration to predict complications.

WBC count in a study by Mickevicius et al. did not differ between the groups. Similar results were presented by Ruiz-Tovar et al. [17], and a study by Da Silva et al. evaluated the correlation between neutrophil-to-lymphocyte ratio (NLR) on POD 1 as a predictive factor for perioperative complications [18]. Da Silva et al.’s study was a retrospective cohort study of 737 patients who underwent predominantly LRYGB, but also LSG. The authors reported that NLR ≥ 10 on POD 1 was associated with the following 30-day clinical outcomes: prolonged hospital stay (> 2 days); higher incidence of overall complications and major complications as well as higher readmission rate; and higher reoperation rate. On multivariable analysis, elevated NLR retained its predictive value for all outcome variables, except for readmissions.

Interestingly, prolonged operative time was a significant predictor for perioperative morbidity in patients undergoing LSG, in our study. We believe that this finding was not a result of longer operation times, but, rather, was secondary to technical difficulties or intraoperative adverse events, which we did not analyze in this study [4, 19]. Major et al. evaluated morbidity after LSG and reported that prolonged operation and increased number of stapler firings were associated with a higher risk of perioperative complications, but interestingly, intraoperative adverse events were not directly associated with an increased risk of perioperative morbidity. In another study by Major et al., decreased oral fluid intake, the need for increased intravenous fluids on the day of surgery, and longer distance from the patient’s habitual residence to the bariatric center were potential risk factors for prolonged hospital stay. Intraoperative adverse events also increased the risk for hospital readmission [4, 19].

In patients undergoing colorectal surgery, measuring inflammatory markers is now almost a routine. However, colorectal surgery has different specificity and higher rates for postoperative morbidity, and leaks are more prevalent, which determines different cut-off points. In a meta-analysis by Cousin et al. of the accuracy of CRP on POD 3 to diagnose intra-abdominal infection after elective colorectal surgery, the cut-offs varied in the studies, from 130 to 190 mg/L [20]. CRP and procalcitonin were significantly higher much earlier than when patients became symptomatic, at between POD 7 and POD 9 [21–23]. This finding is crucial to understand why we performed a similar study in bariatric surgery. Significantly higher CRP levels were found after colorectal surgery as early as POD 1 in patients who later developed intra-abdominal infection, which raises doubts regarding opinions that anastomotic leakage occurs near POD 7 [20, 22, 24]. A stronger inflammatory response may be both the consequence and the cause of intra-abdominal infection (impaired healing or reflecting ongoing tissue hypoxia leading to anastomotic leakage) [21, 25].

The main advantage of our study is the high patient numbers, which makes our conclusions more reliable. We found no ERAS® guidelines indicating laboratory measurements predicting postoperative morbidity. It would be useful to have cut-off points for laboratory measurements of inflammatory markers to select patients requiring longer postoperative stays, for early detection and prevention of postoperative morbidity. Short hospital stays are necessary to maintain a high bariatric surgical volume; therefore, having laboratory criteria for early and safe discharge from hospital would be highly beneficial [26].

### Limitations

This was a non-randomized analysis, and our groups were demographically heterogeneous and differed regarding the preoperative factors. Furthermore, because the data were collected separately from seven bariatric centers using different electronic systems, some necessary information was lacking in our initial population, which caused exclusions. After excluding incomplete records and repetitions in the collective database, we obtained our final number of patients, which added selection bias. Additionally, we did not record postoperative events, such as readmissions occurring outside the indexed hospitals. Patients during hospital discharge were informed to report to index hospital in case of any emergencies. In case of patients that live in a long distance they could refer to local hospitals.

## Conclusion

CRP > 33.32 mg/L and WBC > 12.15 × 10^3^/μL on POD 1 were associated with a greater risk of developing complications in patients undergoing LSG. WBC > 13.78 × 10^3^/μL was associated with a higher risk of morbidity in patients after bypass surgery. Even slightly elevated CRP and WBC on POD 1 should alert surgeons to the potential risk of perioperative morbidity.
